# AI-Written Scientific Manuscripts

**DOI:** 10.3390/tomography11110123

**Published:** 2025-10-30

**Authors:** Emilio Quaia

**Affiliations:** Department of Radiology, University of Padova, Via Giustiniani 2, 35128 Padova, Italy; emilio.quaia@unipd.it; Tel.: +39-049-8212375

This editorial provides insights on AI-written scientific manuscripts which represent an increasingly frequent phenomenon that must be managed by authors, reviewers and journal editors. AI assistance should be accepted as a further tool in scientific manuscript preparation, although some general rules should be proposed and addressed by all authors who are preparing to submit a paper to a specific journal. There is a general need for transparency, accountability, and clear guidelines to ensure that AI serves as a supportive tool rather than a replacement for human scholarly contribution.

AI-written scientific manuscripts are a complex and rapidly evolving area in which AI tools assist in the research and writing process, even though human oversight is essential.

An “AI-written scientific manuscript” is defined as a research paper drafted with the assistance of artificial intelligence tools, offering enhanced efficiency, improved accuracy, and increased clarity, particularly for non-native English speakers. Major journals require disclosure of AI use but prohibit AI from being credited as an author due to ethical concerns over accountability and originality. While AI offers benefits such as enhanced efficiency, improved clarity, and language support, researchers must remain responsible for the accuracy, integrity, and originality of the content. Researchers must verify all AI-generated output, draw their own conclusions, and transparently disclose the use of AI tools in their submissions.

The Committee on Publication Ethics (COPE) currently provides no specific guidance on cases where AI use is implicated in manuscript preparation. However, AI tools cannot meet the criteria for authorship because they cannot be considered responsible for the submitted work. As non-legal entities, AI systems cannot declare conflicts of interest or manage copyright and licensing agreements [1]. Many journals do allow authors to use AI in preparing manuscripts, but transparency is essential. The Ethical Guidelines for Peer Reviewers state that “reviewers should respect the confidentiality of the manuscripts they evaluate. They should not disclose any information about the work or use it for personal advantage, ensuring the integrity of the review process. Reviewers may report in their confidential comments to the editors that the structure and some inconsistencies in the article they are reviewing had raised suspicions that AI algorithms had been used in the preparation of the manuscript. However, if the journal finds that the reviewer’s use of an AI checker has breached the confidentiality of the review process, then the author should be informed. On the other hand, if the editor decides that AI use is implicated in this case they could present it to the author in terms of their policies on transparency, for example, “our policy states any use of generative AI in manuscript preparation should be declared/is prohibited/needs to be described”.

Potential benefits of AI in manuscript writing include the following: AI tools can be used to automate tasks like grammar correction, formatting, and literature review searches, significantly speeding up the manuscript preparation process; AI assistants can improve the readability and grammatical accuracy of papers, which is particularly helpful for non-native English speakers; AI can be used to help ensure that manuscripts adhere to specific journal guidelines, reducing the chance of technical rejection; some AI tools can aid in the analysis and visual representation of data, enhancing the quality of research papers.

The number of papers showing signs of AI use is rising rapidly [2]. Because AI systems can reuse text, they may inadvertently cause plagiarism, raising ethical concerns. Large language models (LLMs) such as ChatGPT can generate and revise text with near-human-level proficiency. To capture a sense of researchers’ views on this topic, *Nature* posed a variety of scenarios to 5000 academics around the world in order to understand which uses of AI are considered ethically acceptable [3]. The survey results suggest that researchers are strongly divided in terms of what they feel are appropriate practices, and academics generally feel that it is acceptable to use AI chatbots to help to prepare manuscripts. According to Kobak et al. [2], among over 15 million biomedical abstracts from 2010–2024 indexed by PubMed, the appearance of LLMs led to an abrupt increase in the frequency of certain style words related to AI-written scientific manuscripts. This excess word analysis suggests that at least 13.5% of 2024 abstracts were processed with LLMs.

As a matter of fact, considering that the number of AI-written scientific manuscripts will increase in future years, this phenomenon should be generally considered acceptable by the scientific community, although some clear rules should be defined. All benefits provided by AI should not justify extended use of AI in scientific writing; instead, AI should be considered a simple additional tool for manuscript preparation. Some recommendations for the use of AI language for scientific communication [4] include the following:

- Disclose the use of AI tools in your manuscript as per the guidelines of the target journal; also, disclose the specific AI tools used and the extent of their involvement in scientific manuscript writing. If generative AI is part of a study’s design, include appropriate methodological detail in the methods section of a manuscript. Describe how generative AI was used in conducting of the scientific work in sufficient detail for a peer-reviewed publication [1]. If generative AI was used to generate manuscript content, then state clearly in the Acknowledgements section how and where the generative AI was used.
- Clearly indicate which sections of the manuscript used the output of the language chatbot.
- The chatbot output should be considered a very early draft and might contain incorrect information; every sentence and statement must be considered critically. Utilize AI simply for tasks like language enhancement, grammar checks, and generating initial drafts, but always review and refine the output.
- Do not list generative AI as a coauthor. AI tools cannot be listed as authors, as they cannot take on the necessary creative, intellectual, or ethical responsibility required of human researchers. Researchers are fully responsible for the accuracy, integrity, and originality of any AI-generated content, and the AI chatbot cannot be held accountable for any statement or ethical breach.
- Be aware that chatbots might also reuse text from other sources and that this may lead to unintentional plagiarism. Consequently, authors must ensure that AI-generated content is not plagiarized and that the final manuscript reflects their own intellectual contributions.
- Chatbots may generate erroneous citations, and any citations recommended by an AI bot/ChatGPT need to be verified. Authors should conduct thorough reviews of AI-generated content to ensure its scientific accuracy and that the meaning remains unchanged.
- The editors should make it clear to reviewers that if they suspect AI use in a paper, they should consult the editor.
- The use of an AI checker for manuscript revision may lead to a breach in the confidentiality of the review process, and this may be appealed against by authors.

## References

[1] Jackson J., Landis G., Baskin P.K., Hadsell K.A., English M. CSE Editorial Policy Committee CSE Guidance on Machine Learning and Artificial Intelligence Tools. Science Editor. 1 May 2023. https://www.csescienceeditor.org/article/cse-guidance-on-machine-learning-and-artificial-intelligence-tools/.

[2] Kobak D., Gonzalez-Marquez R., Horvàt E.A. (2025). Devling into LLM-assisted writing in biomedical publications through excess vocabulary. Sci. Adv..

[3] Kwon D. Is it OK for AI to Write Science Papers? Nature Survey Shows Researchers Are Split. https://www.nature.com/articles/d41586-025-01463-8.

[4] Buriak J.M., Akinwande D., Artzi N., Brinker C.J., Burrows C., Chan W.C.W., Chen C., Chen X., Chhowalla M., Chi L. (2023). Best practices for using AI when writing scientific manuscripts. ACS Nano.

